# 毛细管电泳-激光诱导荧光法测定细胞中谷胱甘肽

**DOI:** 10.3724/SP.J.1123.2022.04018

**Published:** 2023-01-08

**Authors:** Xue MEN, Chengxin WU, Mingli CHEN, Jianhua WANG

**Affiliations:** 东北大学理学院化学系分析科学研究中心, 辽宁 沈阳 110819; Research Center for Analytical Sciences, Department of Chemistry, College of Sciences, Northeastern University, Shenyang 110819, China

**Keywords:** 毛细管电泳-激光诱导荧光, 谷胱甘肽, 砷形态, 铬形态, 细胞毒性, capillary electrophoresis-laser induced fluorescence (CE-LIF), glutathione (GSH), arsenic species, chromium species, cytotoxicity

## Abstract

谷胱甘肽(GSH)在抵抗氧化应激和重金属解毒过程中发挥着重要作用,建立灵敏、准确的GSH定量分析方法对于研究细胞重金属毒性机制具有深远意义。该研究以肝癌细胞(HepG2)为研究对象,以活性基团为芳香邻二醛的2,3-萘二甲醛(NDA)为标记试剂,建立了一种高灵敏度的测定细胞中GSH含量的毛细管电泳-激光诱导荧光检测方法(CE-LIF)。实验考察了缓冲溶液的种类、pH、添加剂等对GSH与NDA的反应速率和NDA-GSH检测灵敏度的影响。比较了pH为7.4和9.2的三羟甲基氨基甲烷(Tris)缓冲溶液、pH为9.2的硼砂和Tris缓冲溶液中NDA-GSH的灵敏度和反应速率,结果显示在pH为9.2的硼砂缓冲溶液中NDA-GSH的灵敏度最高且反应速率最快。进一步比较了4种添加剂对NDA-GSH灵敏度的影响,结果显示以*β*-环糊精(*β*-CD)作为添加剂效果最好。在最优的实验条件下,GSH与NDA可以在5 min内达到反应平衡,3 min内检测到NDA-GSH电泳信号。采用外标法对细胞中的GSH进行定量分析,方法线性范围为0.01~20.00 mmol/L, GSH的检出限和定量限分别为0.006 μmol/L和0.020 μmol/L,加标回收率和标准偏差分别为95.7%~112.6%和3.8%~5.0%(*n*=3)。通过建立的方法对HepG2细胞中的GSH进行定量分析,并研究了As(Ⅲ)、As(Ⅴ)、Cr(Ⅲ)和Cr(Ⅵ)刺激细胞后胞内GSH的变化情况。结果表明,在研究剂量水平下,As(Ⅲ)、As(Ⅴ)和Cr(Ⅲ)不会影响HepG2细胞中GSH的含量,而高剂量Cr(Ⅵ)会导致GSH含量显著降低。结合元素毒性数据,说明HepG2细胞内GSH含量与细胞毒性相关,GSH含量会随着细胞毒性增大而降低。

## Abstract (en)

Glutathione (GSH) is vital for oxidative stress resistance and heavy metals detoxification. It is significant to develop a sensitive and accurate quantitative GSH approach for the toxicity mechanism for studying heavy metals in cells. A high-sensitive capillary electrophoresis-laser induced fluorescence (CE-LIF) detection approach was proposed in this study to detect GSH content in cells. The approach employed HepG2 cells as an object and 2,3-naphthalenedicarboxaldehyde (NDA) with the active group of aromatic *o*-dialdehyde as a labeling reagent. The effects of buffer solution types, pH, additives on the GSH reaction rate with NDA, and the sensitivity of NDA-GSH were systematically investigated. The sensitivity of NDA-GSH and the reaction rate of GSH with NDA were compared in tris(hydroxymethyl)aminomethane (Tris) buffer solution at pH 7.4 or 9.2 and borate-Tris buffer solution at pH 9.2. The results revealed that the NDA-GSH sensitivity was the highest and the reaction rate of GSH and NDA was the fastest in borate buffer solution at pH 9.2. The effects of the four additives on the sensitivity of NDA-GSH were further compared. The best additive was revealed to be *β*-cyclodextrin (*β*-CD). GSH reacted with NDA to reach equilibrium within 5 min under the optimal experimental conditions, and the electrophoretic signal of NDA-GSH could be seen in 3 min. Quantitative analysis of GSH in HepG2 cells was performed using an external standard approach by determining a series of GSH standard solutions. The results revealed that the approach had a good linear relationship with the peak area vs. concentration (0.01-20.00 mmol/L) of GSH. The limit of detection (LOD) and limit of quantification (LOQ) of GSH were determined using signal-to-noise ratios of 3 (*S/N*=3) and 10 (*S/N*=10), which were 0.006 μmol/L and 0.020 μmol/L, respectively. The approach’s spiked recoveries were 95.7%-112.6%, with relative standard deviations of the approach being 3.8%-5.0% (*n*=3). This approach offers high sensitivity, good stability, accuracy, and reliability. To study the relationship between the toxicity of arsenic and chromium on HepG2 cells and the content of GSH in HepG2 cells, the effects of arsenic and chromium with different valences on cell viability were analyzed. The results illustrated that the cytotoxicity of potassium dichromate (Cr(Ⅵ)) was the strongest. The variations of GSH content in HepG2 cells stimulated with arsenite (As(Ⅲ)), arsenate (As(Ⅴ)), chromium chloride (Cr(Ⅲ)), and Cr(Ⅵ) were analyzed by the proposed approach and analysis of intracellular GSH imaging. The results revealed that the stimulation group i. e. analyzed doses (low-dose 2 mg/L, high-dose 5 mg/L) of As(Ⅲ), As(Ⅴ), and Cr(Ⅲ) had no obvious effect on GSH content in HepG2 cells compared with the control group, whereas high-dose Cr(Ⅵ) can significantly reduce GSH content in HepG2 cells. Considering the analysis of cytotoxicity of As(Ⅲ), As(Ⅴ), Cr(Ⅲ), and Cr(Ⅵ), it shows that the content of GSH in HepG2 cells is related to cytotoxicity, and the content of GSH will decrease with the increase in cytotoxicity.

谷胱甘肽(GSH)是一种人体内较丰富的含巯基的三肽化合物,在人体代谢中发挥重要的作用,如解毒作用和抗氧化作用等^[1⇓⇓⇓-5]^。砷(As)和铬(Cr)是工业上常用的两种重金属^[6,7]^,其中As(Ⅲ)和Cr(Ⅵ)毒性较强且会通过呼吸道、消化道等方式进入人体而产生氧化应激反应,从而引起人体内GSH的变化^[8,9]^。人体内GSH的水平变化与多种疾病密切相关^[10]^,如糖尿病、帕金森氏症和阿尔茨海默氏症等,研究发现,脑谷胱甘肽是轻度认知障碍和阿尔茨海默氏症的一种新的生物标志物^[11]^。现在临床上常用的GSH分析检测方法包括电化学法^[12]^、荧光法^[13]^、高效液相色谱-质谱法^[14,15]^、酶分析法^[16,17]^等。

毛细管电泳(CE)是一种具有纳升级样品量和较高分辨率等优点的现代分离分析技术,在生物样品尤其是细胞检测中具有极大的优势。激光诱导荧光法(LIF)是一种灵敏度极高的检测器,因激光易于聚焦,具有高强度和单色性等特点,尤其适合与样品量极少的毛细管电泳联用。CE-LIF联用法具有灵敏度高、样品量小和设备简单易造等特点,尤其适合细胞内GSH的定量分析^[18]^。然而,上述方法中使用了乙腈等有机溶剂,有机溶剂的挥发会导致方法的稳定性较差。

本文构建了毛细管电泳-激光诱导荧光检测系统,优化了缓冲溶液的成分和其他实验条件,建立了一种稳定且灵敏的GSH检测方法。研究了肝癌细胞(HepG2)内GSH含量与金属形态和浓度之间的关系,从结果可以看出,细胞内GSH水平的变化,在一定程度上可以用来评估细胞毒性。

## 1 实验部分

### 1.1 仪器与试剂

构建毛细管电泳-激光诱导荧光检测系统,如图1所示,其中,未修饰的熔融石英毛细管(内径50 μm,外径360 μm,总长度29.5 cm,有效长度25.5 cm,河北永年锐沣色谱器件有限公司)作为分离毛细管。激光诱导荧光检测器为实验室基于共聚焦模式自主搭建^[19]^,其主要参数如下:473 nm激光(20 mW,远明激光,宁波)作为光源,激发光滤光片为470 nm的带通滤光片(汇博光学,沈阳),二向色镜截止波长为500 nm(汇博光学),聚焦物镜为40 X平场物镜(型号NA. 0.6,工作距离3.98 mm,大悦维佳,北京),发射光滤光片为535 nm的带通滤光片(汇博光学),采光针孔孔径为0.4 mm,以光电倍增管(H10722-20,滨松,日本)作为光电探测器进行光信号采集和转换,以数据采集卡(USB-6009, National Instruments,美国)及实验室在LabVIEW平台编写的相关软件进行仪器控制及数据采集。Allegra高速冷冻离心机和HERA Cell 150细胞培养箱分别购自美国Beckman Coulter公司和美国Thermo Scientific公司。SynergyH1酶标仪和BX53M正置显微镜购自美国BioTek公司和日本Olympus公司。KQ-100DB超声波清洗器和08-Ⅲ非接触式超声波细胞破碎仪分别购自江苏昆山市超声仪器有限公司和宁波新芝生物科技股份有限公司。XPE105DR分析天平购自上海梅特勒-托利多国际有限公司。

**图1:**
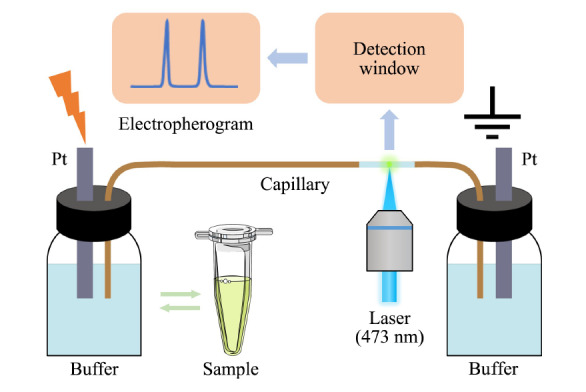
毛细管电泳-激光诱导荧光检测装置示意图

硼砂(borate)、氢氧化钠(NaOH)、十六烷基三甲基溴化铵(CTAB)、*β*-环糊精(*β*-CD)、亚砷酸钠、砷酸氢二钠、氯化铬和重铬酸钾均购自国药集团化学试剂有限公司,2,3-萘二甲醛(NDA)、谷胱甘肽、十二烷基硫酸钠(SDS)、曲拉通X100(Triton X100)、二甲基亚砜(DMSO)、盐酸(HCl)、乙腈(色谱级)和甲醇(色谱级)购自上海阿拉丁生化科技股份有限公司。三羟甲基氨基甲烷(Tris)、达尔伯克(氏)改良伊格尔(氏)(DMEM)培养基、磷酸缓冲盐溶液(PBS)购自美仑生物技术有限公司,胎牛血清、抗生素(链霉素和青霉素)分别购自美国Clark和HyClone公司。HepG2细胞购自武汉普诺赛生命科技有限公司。实验室用水由Milli-Q超纯水系统(美国Millipore公司)制备。除标有纯度的试剂外,以上试剂均为分析纯。

### 1.2 标准溶液配制

准确称取适量GSH固体,溶于超纯水中,配制成10.00 mmol/L的GSH溶液备用,4 ℃避光保存。取适量GSH标准储备液,用超纯水稀释成浓度分别为0.01、0.05、0.20、0.50、1.00、2.00、5.00、20.00 μmol/L的系列标准溶液。

### 1.3 样品预处理

#### 1.3.1 细胞培养

HepG2培养基由50 mL胎牛血清(10%, v/v)、5 mL抗生素(青霉素100 U/mL,链霉素100 μg/mL)加入到450 mL DMEM高糖培养基中混合而成。HepG2细胞于37 ℃、5% CO_2_细胞培养箱中培养。

#### 1.3.2 细胞活力的测定

将HepG2细胞接种在96孔板中贴壁生长,然后加入As(Ⅲ)、As(Ⅴ)、Cr(Ⅲ)和Cr(Ⅵ)溶液(0、2、5、10、20、50和100 mg/L)孵育6 h。用PBS清洗两次,加入100 μL含有0.05 mg/mL噻唑蓝(MTT)的培养基继续孵育4 h。随后,除去培养基,并在每个孔里加入150 μL DMSO(溶解甲瓒晶体),使用酶标仪在570 nm处测量每孔的吸光度。

#### 1.3.3 砷和铬孵育的细胞样品制备

将HepG2细胞分别接种在25 cm^2^的培养瓶中以贴壁生长,再加入As(Ⅲ)、As(Ⅴ)、Cr(Ⅲ)和Cr(Ⅵ)溶液(2和5 mg/L)孵育6 h,同时设置空白对照组。用PBS清洗3次后,收集细胞,冻干后,加入100 μL背景缓冲溶液,超声裂解后进行超速离心(12000 r/min, 15 min),收集细胞裂解液并存放于-80 ℃备用。

#### 1.3.4 GSH的标记和胞内成像

标记GSH:采用文献报道的GSH标记方法^[20]^,并对该方法进行了优化。在背景缓冲溶液(20 mmol/L硼砂和2 mmol/L *β*-CD, pH 9.2)中加入1 μL GSH标准溶液(10 μmol/L)和细胞裂解液,然后加入100 μmol/L的NDA溶液,反应5 min后,样品注入分离毛细管。

胞内GSH成像:将HepG2细胞分别接种在96孔板中贴壁生长,再加入5 mg/L As(Ⅲ)、As(Ⅴ)、Cr(Ⅲ)和Cr(Ⅵ)溶液孵育6 h。经PBS清洗2次后,每个孔里加入200 μL 1 mmol/L的NDA溶液,室温下避光孵育10 min。经PBS清洗3次以终止染色。最后,在每个孔里加入500 μL PBS,在正置显微镜下观察和记录,放大倍数为10倍。

### 1.4 实验条件与数据处理

#### 1.4.1 CE-LIF条件

毛细管在使用前依次用甲醇、超纯水、0.1 mol/L NaOH、超纯水、0.1 mol/L HCl、超纯水各冲洗10 min。每次进样前,用0.1 mol/L NaOH、超纯水和背景缓冲溶液冲洗1 min。重力进样,抬高10 cm,进样10 s,室温20 ℃下,阳极进样,阴极检测,电压为14 kV,激发波长为473 nm,发射波长为528 nm。背景缓冲溶液、GSH标准溶液和细胞裂解液在电泳之前需经0.22 μm微孔滤膜(水系)过滤,并超声脱气5 min。

#### 1.4.2 数据处理

GSH的定量数据通过实验室的电泳分析软件计算峰面积后,再用Excel和GraphPad Prism进行绘图和统计分析。电泳图直接由Origin Pro2016绘制。

## 2 结果与讨论

### 2.1 电泳条件优化

#### 2.1.1 缓冲溶液种类和pH值的影响

电泳缓冲溶液对标记反应和电泳性能起着至关重要的作用。为了选择合适的缓冲溶液,使用酶标仪考察了在20 mmol/L的硼砂和Tris两种缓冲溶液(Tris pH 7.4和pH 9.2,硼砂pH 9.2)下GSH(10.00 μmol/L)与NDA(100 μmol/L)的反应动力学。每个条件运行时间30 min,时间间隔1 min,激发波长设为473 nm,发射波长设为528 nm,检测高度为4.4 mm。

结果如图2所示,在硼砂缓冲液中(pH 9.2), NDA自身不发荧光,当加入GSH后,NDA衍生GSH产生荧光产物^[20]^,在5 min内达到反应平衡且在30 min内保持稳定,而在Tris缓冲液中(pH 9.2), GSH与NDA反应在25 min达到平衡,平衡时NDA-GSH的荧光强度也弱于在硼砂缓冲液(pH 9.2)中的荧光强度。在pH为7.4的Tris缓冲液中,反应平衡时间相较于在pH为9.2的Tris缓冲液中缩短10 min,但荧光强度较弱。考虑到反应时间和荧光强度,选择pH为9.2的硼砂缓冲液作为GSH电泳分析的缓冲液。

**图2:**
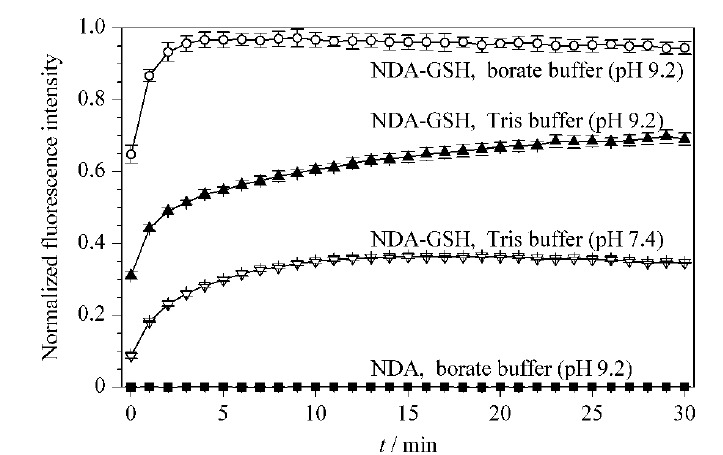
背景缓冲液对GSH与NDA反应动力学的影响(*n*=3)

#### 2.1.2 添加剂的选择和条件优化

为了在保证电泳稳定性的同时提高NDA-GSH的灵敏度,使用酶标仪考察了在20 mmol/L硼砂缓冲液中加入10 mmol/L SDS、5 mmol/L CTAB、5 mmol/L Triton X100和10 mmol/L *β*-CD时NDA-GSH的灵敏度。图3a和3b中,反应动力学的参数设置与2.1.1节相同,NDA-GSH的发射光谱是NDA与GSH衍生5 min后,在激发波长为473 nm下获得的。结果表明,在硼砂缓冲液中加入以上4种添加剂后,NDA-GSH的灵敏度都有所提高,NDA-GSH的发光特性没有明显改变,发射波长保持在528 nm左右。当CTAB和*β*-CD加入后,NDA-GSH的灵敏度明显提高。SDS和Triton X100的加入也对提高NDA-GSH的灵敏度有所帮助,但是平衡时间在10 min内。综合考虑反应时间和灵敏度,进一步在缓冲液中加入CTAB和*β*-CD进行电泳实验。如图3c所示,当加入CTAB后,没有样品峰出现,可能是CTAB作为一种阳离子型表面活性剂,改变了电渗流的方向。当加入*β*-CD后,NDA-GSH的信号明显增强,迁移时间也缩短至3 min内。于是,进一步考察了*β*-CD的浓度对NDA-GSH灵敏度的影响。随着*β*-CD浓度的增加,NDA-GSH的迁移时间缩短,其信号强度先增大后减小,在*β*-CD浓度为2 mmol/L时,NDA-GSH的信号最强(见图3c)。因此,最终使用的背景缓冲溶液组成为20 mmol/L硼砂和2 mmol/L *β*-CD的混合溶液。

**图3:**
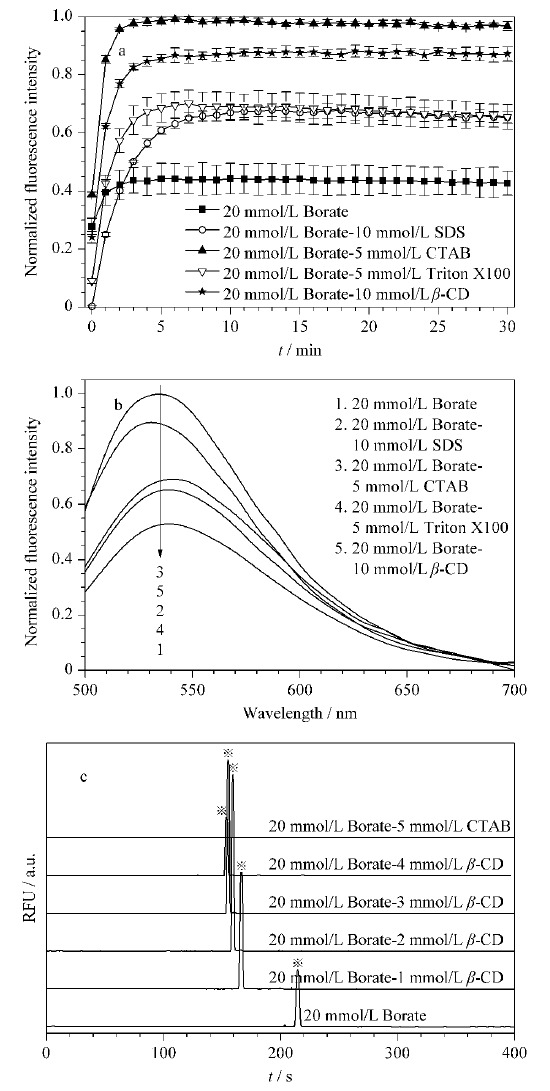
硼砂缓冲液中的添加剂对NDA-GSH灵敏度的影响

### 2.2 方法性能

#### 2.2.1 分析性能

采用1.2节的方法,制备0.01、0.05、0.20、0.50、1.00、2.00、5.00、20.00 μmol/L的GSH标准溶液,在优化的条件下,每份溶液平行3次,用实验室的电泳分析软件对峰面积积分,以峰面积为纵坐标(*y*), GSH的浓度为横坐标(*x*, μmol/L),绘制标准曲线。结果表明,GSH在0.01~20.00 μmol/L范围内,与峰面积呈现良好的线性关系,回归方程为*y*=0.21*x*+0.0004,相关系数为0.9993。以信噪比为3(*S/N*=3)和10(*S/N*=10)确定GSH的检出限和定量限,分别为0.006 μmol/L和0.020 μmol/L。与现有的文献^[20]^相比,该方法GSH的检测灵敏度提高了约40倍。

#### 2.2.2 准确度和精密度

以稀释200倍的空白细胞裂解液为基质进行低、中、高3个水平的加标回收试验,加标浓度分别为0.10、1.00、10.00 μmol/L,在优化的条件下,每个水平平行测定3次。结果如表1所示,GSH的回收率为95.7%~112.6%,精密度为3.8%~5.0%,表明该方法的准确性和重复性良好,证明了其在细胞内GSH分析中的可靠性。

**表1:** GSH在细胞裂解液中的加标回收率和精密度(*n*=3)

Analyte	Background/ (μmol/L)	Added/ (μmol/L)	Found/ (μmol/L)	Recovery/ %	RSD/ %
GSH	0	0.10	0.09	99.2	5.0
	0	1.00	0.96	95.7	4.0
	0	10.00	11.26	112.6	3.8

### 2.3 砷和铬对HepG2细胞内GSH水平的影响

#### 2.3.1 细胞活性和形态

为了研究砷和铬对HepG2细胞产生的毒性大小与细胞内GSH含量的关系,首先考察了不同价态的砷和铬对细胞的活性影响(见图4)。从图4可知,在研究的浓度范围内,As(Ⅲ)和Cr(Ⅵ)对细胞的毒性随浓度的增加逐渐增大,而As(Ⅴ)和Cr(Ⅲ)没有表现出对细胞有明显的毒性。质量浓度为5 mg/L的As(Ⅲ)、As(Ⅴ)、Cr(Ⅲ)和Cr(Ⅵ)对细胞刺激后的细胞活性分别为96%、98%、96%和89%。4种元素形态对细胞产生的毒性大小顺序为:Cr(Ⅵ)>As(Ⅲ)≈Cr(Ⅲ)>As(Ⅴ)。

**图4:**
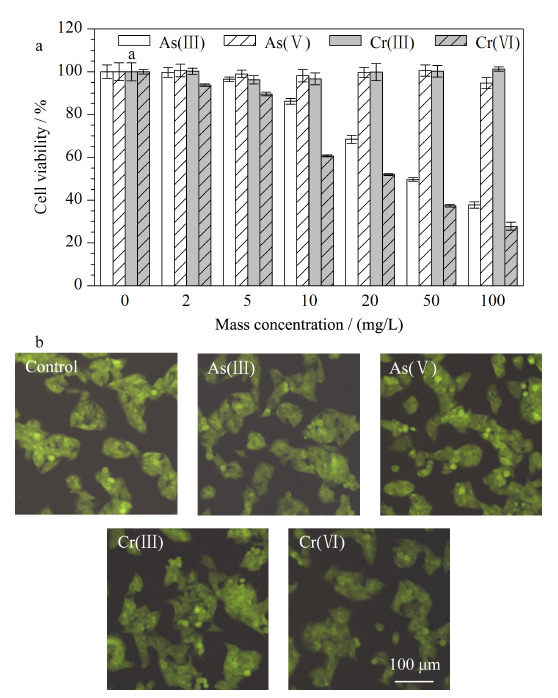
(a)HepG2细胞的细胞活性(*n*=4)和(b)胞内GSH成像

考察了2 mg/L(低剂量)和5 mg/L(高剂量)的4种元素形态对细胞内GSH含量的影响。根据文献^[21]^报道,细胞内GSH的含量随着暴露金属浓度的增加而减少。因此,考察了高剂量的4种元素刺激细胞时的细胞成像效果。如图4所示,以高剂量刺激细胞仍然可以检测到NDA-GSH的荧光信号,表明以高剂量刺激细胞确保了细胞内可检测的GSH,并且不会影响细胞的形态。另外,通过Image J软件进行平均荧光强度分析,5 mg/L的As(Ⅲ)、As(Ⅴ)、Cr(Ⅲ)和Cr(Ⅵ)对细胞刺激后的胞内NDA-GSH的平均荧光强度值依次为69.9、54.1、62.6、54.7和48.5。平均荧光强度值越大,说明GSH含量越高。Cr(Ⅵ)刺激后胞内的NDA-GSH荧光强度最低,说明胞内GSH含量最低,并且Cr(Ⅵ)的细胞毒性最强。初步表明细胞毒性越强,其胞内GSH含量越低。

#### 2.3.2 砷和铬的形态和浓度对HepG2细胞内GSH含量的影响

用构建的毛细管电泳-激光诱导荧光系统研究了砷和铬的形态和浓度对HepG2细胞内GSH含量的影响。以低剂量和高剂量As(Ⅲ)、As(Ⅴ)、Cr(Ⅲ)和Cr(Ⅵ)刺激HepG2细胞6 h后,在优化的电泳条件下,胞内的GSH含量和细胞裂解液电泳结果如图5所示,通过与标准品电泳谱图的迁移时间比对和标准加入法来鉴别细胞裂解液中的GSH。通过统计学分析,相比对照组,以低剂量和高剂量的As(Ⅲ)、As(Ⅴ)和Cr(Ⅲ)刺激细胞,胞内GSH的含量变化没有显著差异。然而,相比对照组,以高剂量Cr(Ⅵ)刺激细胞,胞内GSH的含量显著降低(*P*<0.01),并且胞内GSH的含量与以高剂量Cr(Ⅲ)刺激细胞时具有明显差异(*P*<0.05)。这进一步表明了细胞毒性越大,胞内GSH的含量越低。

**图5:**
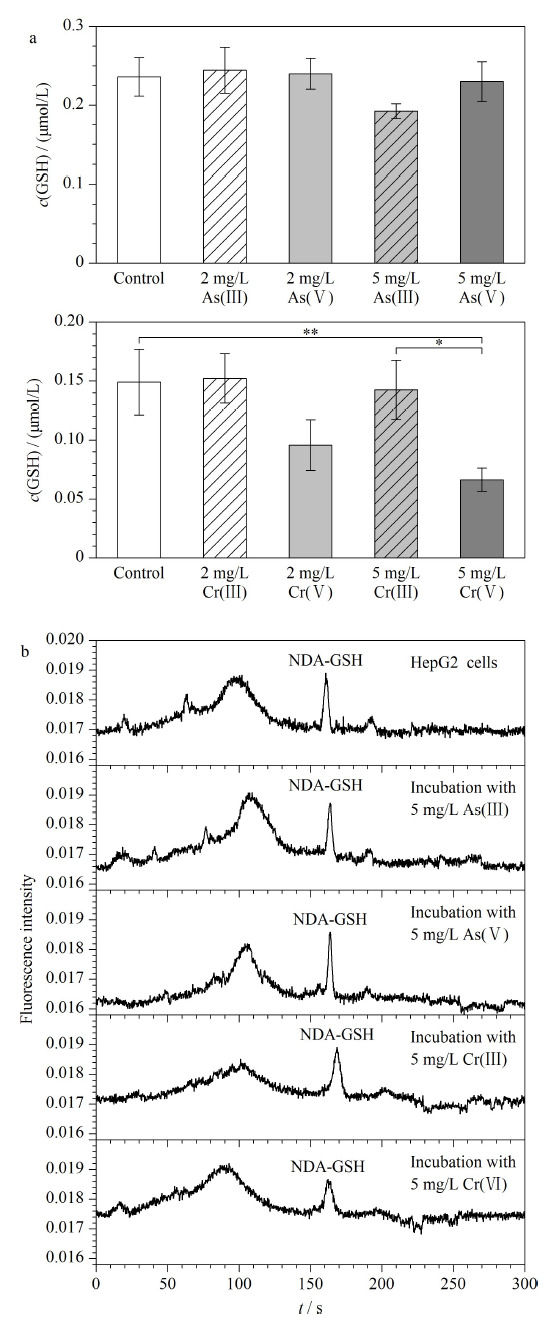
(a)HepG2细胞内的GSH含量的统计分析(*n*=3)和(b)细胞裂解液的电泳图

## 3 结论

本研究建立了一种毛细管电泳-激光诱导荧光检测HepG2细胞中GSH含量的方法。通过该方法研究了砷和铬的形态和浓度对HepG2细胞内GSH含量的影响,以毒性较强的高剂量Cr(Ⅵ)刺激细胞导致胞内GSH含量显著降低,表明胞内GSH含量与细胞毒性相关,即细胞毒性越大,胞内GSH的含量越低。该方法灵敏度高,稳定性好,准确度高,为重金属的浓度监测和评估重金属对细胞的毒性影响提供了新的手段。
